# Multimodal Analysis of Child-Horse Therapeutic Interactions Using Vision-Language Models in an Australian Aboriginal Equine-Assisted Learning Program for Social and Emotional Well-Being: Development and Evaluation Study

**DOI:** 10.2196/95887

**Published:** 2026-10-30

**Authors:** Karma Dechen Dema, Juli Coffin, Sharmila Vaz, Guanjin Wang, Craig Olsson

**Affiliations:** 1 School of Information Technology Murdoch University Murdoch, Western Australia Australia; 2 School of Population Health Curtin University Perth, Western Australia Australia; 3 The Centre for Social Impact The University of Western Australia Perth, Western Australia Australia; 4 Deakin Lifespan Institute, School of Psychology, Faculty of Health Deakin University Burwood, Victoria Australia; 5 Melbourne Children’s LifeCourse Group, Murdoch Children’s Research Institute Royal Children’s Hospital Campus Parkville, Victoria Australia

**Keywords:** child and adolescent health, digital health, EAL, equine-assisted learning, human-animal interaction, multimodal AI, vision-language models, VLMs, youth well-being

## Abstract

**Background:**

The human-horse bond has played a role in human development throughout history and has more recently been applied to mental health promotion through equine-assisted learning (EAL) programs. In EAL settings, therapeutic documentation and assessment commonly rely on practitioner observations documented through behavioral checklists, structured interviews, and progress notes, which can pose challenges due to time demands, subjectivity, and inconsistent standardization across practitioners and contexts.

**Objective:**

This study aimed to address these challenges in the context of mental health promotion programs. This study developed an AI-powered digital health framework based on vision-language models (VLMs) to analyze child-horse therapeutic interactions in EAL settings. The framework was developed in collaboration with the Yawardani Jan-ga EAL program, which supports the social and emotional well-being of Aboriginal youth in the Kimberley region of Western Australia.

**Methods:**

The proposed framework comprises three core components: (1) an activity classification module using a VLM with few-shot learning based on Sigmoid Loss for Language-Image Pre-training 2 (SigLIP2) to identify 9 therapeutic activities; (2) a proxemics analysis module using a Qwen2.5-VL-7B VLM to classify physical contact between the child and horse; and (3) a body posture analysis module applying multimodal chain-of-thought prompting and self-consistency techniques to classify children’s body posture into confidence-related states with interpretable reasoning.

**Results:**

The framework was developed and evaluated using real-world images collected during therapeutic sessions with Aboriginal children participating in the Yawardani Jan-ga program. The activity classification module achieved an accuracy of 90.6%, with macroaveraged precision, recall, and *F*_1_-scores of 92.0%, 91.1%, and 91.1%, respectively, under the selected 2-shot configuration. The proxemics analysis module achieved an accuracy of 88.6%, with macroaveraged precision, recall, and *F*_1_-scores of 89.0%, 88.0%, and 88.5%, respectively, under the 1-shot configuration. For the body posture analysis module, system reliability was assessed using internal consistency metrics, achieving an average consistency of 0.916, an agreement rate of 0.705, a rationale coherence of 0.857, and a vote entropy of 0.174.

**Conclusions:**

Overall, the findings suggest that AI- and VLM-based approaches can provide complementary support for practitioner-led documentation and assessment of observable child-horse therapeutic interactions in EAL settings. These approaches may help reduce practitioner burden and strengthen the assessment of programs that promote children’s social and emotional well-being.

## Introduction

### Overview

The relationship between humans and horses has long been recognized as a powerful source of emotional healing and psychological support. Research demonstrates that equine-assisted interventions, including equine-assisted learning (EAL) programs, have shown promise in supporting the mental health and emotional development of children and adolescents [1]. Through structured human-horse interactions, participants often experience reduced anxiety and depression, improved self-esteem, greater emotional resilience, and better quality of sleep [2-4]. These therapeutic relationships create powerful opportunities for healing and growth in structured therapeutic settings. These programs also foster a sense of emotional safety, personal security, and connectedness, particularly for at-risk children and young people who may struggle with low self-esteem or unstable learning environments [5,6]. Central to these outcomes is the horse’s unique socioemotional intelligence, which enables deep, nonverbal communication and trust-building with humans [7], making EAL a powerful tool for healing and growth in therapeutic settings. The Yawardani Jan-ga (“horse helping”) [8] project in Western Australia’s Kimberley region exemplifies this approach by integrating evidence-based EAL methodologies within a culturally secure, community-led framework. The program works with Aboriginal children and young people aged 6-25 years and is designed to respond to the unique educational and social challenges faced by Indigenous youth in remote communities. The program supports educational engagement and achievement by addressing underlying social and emotional well-being barriers that often hinder learning and participation [9,10].

Traditional evaluation tools in EAL settings rely on behavioral observations, structured interviews, standardized questionnaires, and physiological measurements conducted during sessions [11,12]. Despite their widespread use, these approaches present several limitations, including challenges with standardization, high time demands, and vulnerability to observer bias, which could lead to low interrater reliability across practitioners [1]. Physiological monitoring methods, such as heart rate or cortisol analysis, can provide objective indicators; however, the required equipment may be intrusive and risk disrupting natural child-horse interactions, potentially influencing the behaviors being measured [13]. There remains a lack of standardized, noninvasive tools that can objectively support the evaluation of processes and outcomes in EAL programs [14,15].

In practice, photographs are frequently collected during EAL sessions to capture key moments of child-horse interaction, supporting reflection, storytelling, practitioner documentation, and evaluation activities [16]. However, there has been limited research on systematic methods for analyzing such image-based data, despite its potential to provide complementary, evidence-informed insights into interaction patterns, emotional responses, and subtle behavioral cues that may not be readily captured through traditional observational methods. With advances in computational power and data storage capability, AI techniques have evolved significantly over the past decade. These techniques are now widely applied to a range of computer vision tasks, including image classification, object detection, and scene understanding [17-19]. More recently, vision-language models (VLMs) have emerged as a breakthrough in AI. Trained on large-scale datasets comprising paired images and text, VLMs bridge computer vision and natural language processing, enabling models to understand and generate natural language descriptions of visual content [20]. The ability of VLMs to generate text-based explanations from visual data offers new opportunities for more interpretable image-based analysis, facilitating human judgment and downstream analytical tasks. For example, in medical imaging, VLMs trained on extensive clinical datasets have enabled applications such as automated medical report generation from radiological scans and pathology slides, visual question answering for extracting diagnostic insights from medical images, and multimodal analysis combining text and imaging data for more comprehensive patient assessment. These applications demonstrate VLMs’ ability to interpret complex visual information and generate clinically relevant insights [21,22]. However, applications of AI to human-animal interactions in EAL settings remain limited. Existing studies have primarily focused on AI-based systems for monitoring equine behavior in broader contexts [23], with little research exploring the use of VLMs to analyze child-horse interactions in EAL programs.

Motivated by these gaps, we developed a VLM-assisted AI framework for analyzing images collected during EAL activities, in collaboration with the Aboriginal-led and culturally secure Yawardani Jan-ga EAL program in Western Australia, which supports the social and emotional well-being of Aboriginal children and young people. Training and testing datasets were curated from images collected during EAL sessions within the program. Co-designed with program domain experts and certified EAL practitioners, the framework uses VLMs to achieve three analytical objectives: (1) activity classification: automatically identify and categorize 9 therapeutic activities (eg, grooming and leading) from EAL session images; (2) proxemics analysis: analyze spatial relationships between children and horses by assigning proximity categories to document observed engagement patterns; and (3) body posture analysis: describe and classify children’s body language and postural indicators through transparent and interpretable reasoning. To the best of our knowledge, this is the first study to apply VLMs to child-horse interactions in EAL settings, combining few-shot learning, generative reasoning, and multimodal chain-of-thought (MCoT) prompting, and self-consistency to support interpretable image analysis with minimal labeled data. The framework provides structured outputs through 3 parallel analytical modules, which could assist EAL practitioners in documenting and reviewing observable interactions across activity types, proximity patterns, and body postures. By investigating specialized VLM-based image analysis tools in EAL settings, this study illustrates the potential of AI- and VLM-based approaches to complement practitioner-led documentation and support more structured analysis of child-horse therapeutic interaction patterns in programs that promote the social and emotional well-being of Aboriginal youth.

### Related Work

This section reviews research areas relevant to the design of our framework for analyzing child-horse interactions in EAL settings.

#### VLMs

VLMs are AI systems designed to bridge the gap between visual perception and linguistic reasoning by learning from massive datasets of image-text pairs [24]. By jointly modeling visual and textual information, VLMs capture rich semantic knowledge that enables interpretation of complex visual scenes [20,24,25]. Early foundational approaches, such as Contrastive Language-Image Pre-training (CLIP) [24], revolutionized the field by aligning visual and textual representations in a shared embedding space through contrastive learning and enabling 0-shot learning. Building on this paradigm, Sigmoid Loss for Language-Image Pre-training (SigLIP) refined image-text alignment by replacing contrastive loss with a sigmoid-based loss, aiming to achieve better scalability and fine-grained alignment [26,27]. While earlier VLMs primarily focused on learning aligned visual-textual representations, the introduction of visual instruction tuning, pioneered by models such as the Large Language-and-Vision Assistant (LLaVA), transformed VLMs into interactive systems capable of following complex user instructions grounded in visual inputs [28]. LLaVA demonstrated that projecting visual features into the large language model token space enables instruction-following, reasoning, and visual question answering based on image content. However, these initial architectures were structurally limited to processing single images. More recent models, including Qwen-VL and Qwen2.5-VL, further advanced this architecture by supporting dynamic image resolution handling, flexible aspect ratios, multi-image processing, and temporal understanding [29,30]. Similarly, LLaVA-OneVision has overcome the single-image restriction by introducing a unified framework that seamlessly handles multi-image and video scenarios [31,32]. Computer vision techniques have been used to monitor equine behavior in general contexts [23], including the recognition of behaviors such as locomotion, feeding, drinking, resting, and other stall-based behaviors [33,34]. In addition, broader welfare-monitoring systems have integrated AI to detect abnormal patterns early [35]. While these studies demonstrate the growing potential of AI for analyzing horse behavior, they have largely focused on horse-centered behaviors rather than the interactive and relational dynamics central to EAL. In EAL settings, value often emerges through embodied interaction and the development of human-horse relationships [7]. In parallel, VLMs have been applied to human-centered domains, and the interpretation of body posture has primarily relied on vision-based deep learning methods. However, the use of VLMs to analyze child-horse interactions in EAL settings remains largely unexplored.

#### Few-Shot Learning

Few-shot learning is a technique that allows AI models to perform new tasks using only a small number of examples, rather than requiring large-scale training data [36,37]. In the context of VLMs, this is often implemented as in-context learning, where the model is “guided” by a few example demonstrations provided directly within the prompt [37]. This approach is particularly valuable in specialized domains such as health care, where labeled data are often scarce or expensive to obtain [38]. By adapting pretrained models via prompting rather than costly parameter updates, few-shot learning enables the recognition of domain-specific behaviors using minimal sample sizes. Recently, in-context learning has been applied to VLMs for medical imaging applications. Ferber et al [39] demonstrated that GPT-4V, equipped with in-context learning, can compete with or even outperform specialized neural networks on cancer histopathology image classification benchmarks in terms of accuracy, using only 5 to 10 example images per class label. Similarly, Agbareia et al [40] observed that few-shot prompting leads to substantial improvements in diagnostic accuracy for retinal disease classification of optical coherence tomography images, indicating that in-context learning is a practical approach for applying VLMs in medical applications where annotated data remains limited.

#### Chain-of-Thought Reasoning

Chain-of-thought (CoT) prompting is a technique that guides AI models to break down complex reasoning into explicit step-by-step explanations [41]. Rather than producing direct answers, CoT models display their reasoning process, allowing verification that the AI arrived at its conclusion logically. More recent work has extended CoT reasoning to multimodal contexts where both visual and textual information must be processed together. Zhang et al [42] introduced MCoT reasoning, which separates the reasoning process into 2 stages: first, generating rationales that describe observable visual features, then using these rationales to make final predictions. This 2-stage approach reduces errors by forcing models to explicitly ground their judgments in visual observations rather than jumping directly to conclusions. Wang et al [43] demonstrated that self-consistency significantly improves CoT reasoning. Instead of relying on a single CoT output, self-consistency runs the model multiple times with temperature-based sampling, allowing exploration of different reasoning paths. The final answer is determined by majority voting across these multiple runs, with consensus strength indicating the reliability of the prediction. This combination enhances reliability by ensuring that decisions are first grounded in explicit visual evidence to minimize hallucination, and then statistically verified through repetition to filter out stochastic errors. In general, CoT prompting has shown promise for improving clinical reasoning in VLMs. Zero-shot CoT prompting has been used to make the diagnostic reasoning of VLMs in medical visual question answering more explicit and clinically interpretable [44]. Other multimodal CoT frameworks for medical imaging, such as models that generate vision-to-text CoT explanations for diagnosis [45] and structured visual CoT pipelines for medicine [46], trace stepwise reasoning from image findings to diagnostic conclusions, helping reduce hallucinations. These approaches suggest that CoT prompting can improve both the transparency and reliability of AI-assisted clinical decision-making in health care settings.

### Research Gap and Framework Rationale

Building on prior work, our framework integrates VLMs, few-shot learning, and MCoT reasoning to address key tasks that facilitate the analysis of EAL interactions by enabling visual interaction analysis, supporting practical use with limited labeled data, and promoting interpretable and reliable reasoning grounded in visual evidence.

## Methods

### Framework Architecture

We propose a new VLM-based framework for understanding human-horse interactions in EAL using images and expert-verified label descriptions. The framework was developed in collaboration with the Yawardani Jan-ga program and co-designed with EAL practitioners and researchers to ensure alignment with practice-based needs. The Yawardani Jan-ga program integrates structured learning activities involving horses, such as grooming and leading, to support the social and emotional well-being of Aboriginal children and young people. The program seeks to capture human-horse interactions by identifying critical observable interactions through analyses of photo and video footage collected during EAL sessions, to quantify and rate equine-human dyadic interactions using coding schemes [10]. To support the analytic objective, the framework consists of three core learning modules: (1) an activity classification module that uses a VLM with few-shot learning to identify 9 therapeutic activities; (2) a proxemics analysis module that classifies spatial relationships between the child and horse, including physical contact; and (3) a body posture analysis module that applies MCoT prompting and self-consistency techniques to classify children’s body posture into confidence-related categories with transparent reasoning. The overall framework is illustrated in Figure 1. To address practical requirements related to data privacy and deployment feasibility in remote EAL service areas where network connectivity may be limited, all VLMs used in this framework were selected from open-source models that support local deployment. The following subsections describe each module in detail.

**Figure 1:**
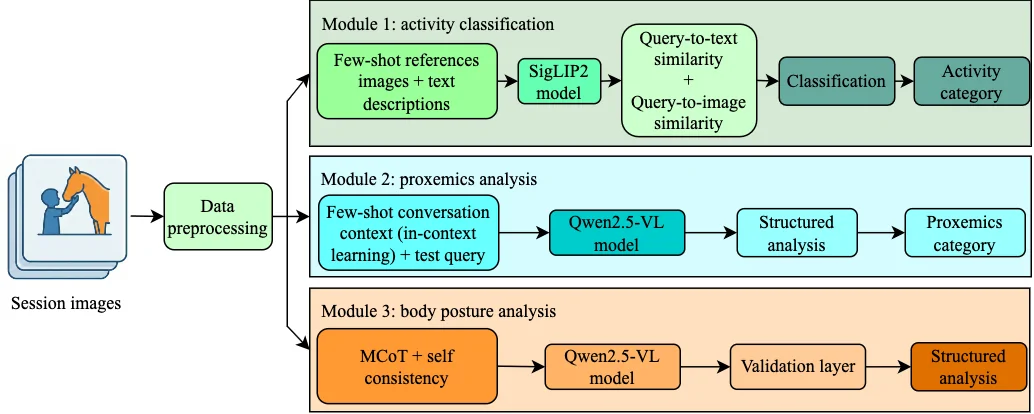
Overview of the proposed vision-language model–assisted AI framework for analyzing child-horse interactions in the Yawardani Jan-ga equine-assisted learning program. The framework comprises three core modules: (1) an activity classification module using Sigmoid Loss for Language-Image Pre-training 2 (SigLIP2), an image-text alignment model; (2) a proxemics analysis module using Qwen2.5-VL-7B, a generative vision-language model; and (3) a body posture analysis module using Qwen2.5-VL-7B with multimodal chain-of-thought reasoning and self-consistency.

### Activity Classification Module

The Yawardani Jan-ga EAL program is structured around 10 therapeutic themes (eg, noticing, awareness, boundaries, and healthy relationships) grounded in the neurosequential model of therapeutics principles [10]. Each theme encompasses multiple activities, and a single activity can serve more than one theme. For this study, 9 representative activities were selected in consultation with EAL practitioners for this activity classification module. Below is a textual description of each activity.

- Ball activities: a photograph of a young person and a horse with a giant ball in sight outdoors. The person is engaging the horse with the ball, a young person is sitting on the giant ball, or a young person is playing with the ball.
- Grooming: a photograph of a young person carefully brushing a horse’s coat. The person is using grooming tools to clean and take care of the horse.
- Herd observation: a photograph of a young person observing a horse or multiple horses from a distance, typically with a fence or barrier between them. The person may be sketching or drawing the horses while watching them.
- Leading the horse: a photograph of a young person leading a horse with a rope or lead line on an open path. The person is walking beside or in front of the horse, guiding it with a rope on clear ground. There are no cones, no poles, no barriers, no tires, no balls, and no obstacles in their path.
- Leading with obstacles: a photograph of a young person guiding a horse through an obstacle course. The person is leading the horse around cones, poles, barriers, barrels, or tires, not a ball.
- Mounted sessions: a photograph of a young person mounted on horseback. The person is sitting atop the horse.
- Painting: a photograph of a young person applying paint to a horse’s body with a paintbrush. The person is decorating the horse with colorful paint designs.
- Touching: a photograph of a young person gently touching a horse with their bare hands. The person is making gentle physical contact with the horse’s body with their bare hands, no tools used.
- Washing the horse: a photograph of a young person washing a horse with water and possibly soap. The person is using water to clean the horse’s coat.

Activity classification in EAL presents unique challenges, as different therapeutic activities may exhibit similar visual features while differing substantially in therapeutic intent and clinical relevance. For example, grooming and touching may both visually appear as a child’s hand in contact with a horse; however, grooming typically involves using tools such as brushes, whereas touching involves direct hand contact. These subtle yet meaningful distinctions motivate the use of VLMs that can align visual features with semantically precise textual descriptions to support more accurate activity interpretation. Accordingly, we adopted an image-text similarity–based approach using Sigmoid Loss for Language-Image Pre-training 2 (SigLIP2; google/siglip2-giant-opt-patch16-384) [26] as the foundational visual-linguistic embedding model. SigLIP2 provides strong alignment between visual and textual representations, supports fine-grained visual semantics, and demonstrates effectiveness in 0-shot and few-shot scenarios, making it well-suited for EAL settings characterized by limited labeled data and context-dependent activity definitions. We compared SigLIP2 against OpenAI’s CLIP-ViT-L-14 [24] to validate this model.

As illustrated in Figure 2, the activity classification module operates in 2 phases. During initialization, predefined textual descriptions for 9 activity categories are encoded using SigLIP2’s text encoder, and a small number of reference images per activity category are encoded using SigLIP2’s vision encoder and averaged to form visual prototypes. The text and image encoders produce embeddings that are aligned in a shared semantic space, enabling direct cosine similarity computation between visual and textual representations. In the classification phase, a query image is encoded using the same vision encoder and compared against both textual and visual prototype representations using cosine similarity. To integrate semantic and visual evidence, we introduce a weighted fusion strategy, defined as:



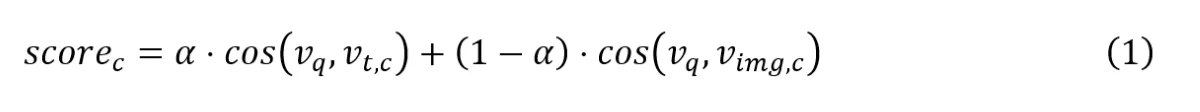



where *score_c_* denotes the score for each activity class *c*, *v_q_* represents the visual embedding of the query image. *v_t,c_* represents the textual embedding of the predefined description for the activity category *c*. *v_i_*_mg,_*_c_* denotes the visual prototype for class *c* calculated as the mean embedding of the *n* reference images provided during the initialization phase, and α controls the relative contribution of textual and visual similarities. The value of α was tuned separately using grid search over values from 0.0 to 1.0 in increments of 0.1 on the validation set, with macro *F*_1_-score used as the selection criterion. Finally, softmax normalization yields probability distributions and confidence scores for different activities.

**Figure 2:**
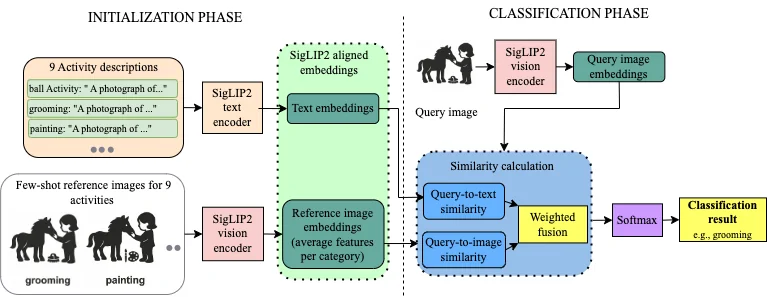
Pipeline of the activity classification module in the proposed vision-language model–assisted AI framework for analyzing child-horse interaction images from the Yawardani Jan-ga equine-assisted learning program. The module uses Sigmoid Loss for Language-Image Pre-training 2 (SigLIP2) to encode predefined textual descriptions and few-shot reference images of 9 therapeutic activities into an aligned embedding space, then classifies query images by combining text-image and image-prototype cosine similarity scores.

### Proxemics Analysis Module

The proxemics analysis investigates spatial relationships between children and horses during therapeutic interactions. Interactions are classified into two therapeutically meaningful categories: (1) “physical contact,” where the child directly touches the horse through hands, arms, or body; (2) “no physical contact,” where the child is positioned near the horse without direct physical touch. Prior work has shown that contrastive VLMs, such as CLIP and SigLIP, have limited capacity to model spatial relationships [47], and our preliminary experiments similarly indicated that these models struggled to reliably distinguish contact-based interactions in our study settings. Following initial testing, we adopted the open-source Qwen2.5-VL-7B-Instruct model for proxemics analysis. Unlike contrastive VLMs, which rely on embedding similarity, Qwen2.5-VL is a generative VLM that performs joint image-text reasoning, enabling more explicit interpretation of spatial relationships and interaction context [29]. The 7B parameter configuration was selected because smaller models did not consistently support coherent multistep reasoning, whereas larger variants offered only limited performance gains at substantially higher computational cost [48].

As illustrated in Figure 3, the framework leverages few-shot in-context learning by pairing each preconfigured proximity category with expert-validated reference examples. For each query image, a structured multimodal template is constructed by combining the n-shot reference examples with the query image. The reference examples are formatted as user-assistant dialogue pairs that demonstrate correct interpretations for each category, followed by the query image and a task-specific prompt instructing the model to classify the interaction. The model jointly processes the reference and query images alongside the text instructions, reasoning over the visual content and instructional context to generate its response. The model generates a free-form natural language response describing the observed interaction. This output is then parsed to extract the primary binary label (physical contact vs no physical contact). When physical contact is identified, the module extracts the number of hands involved in the contact, such as 1 hand or 2 hands. When no physical contact is present, the module extracts the estimated distance between the child and the horse, such as very close or far away. Hand count and estimated distance are descriptive subattributes intended to provide additional contextual information for practitioners. The primary evaluated output of the proxemics module is the binary contact classification. We compared Qwen2.5-VL-7B-Instruct against LLaVA-OneVision-7B to further evaluate the proxemics classification task.

**Figure 3:**
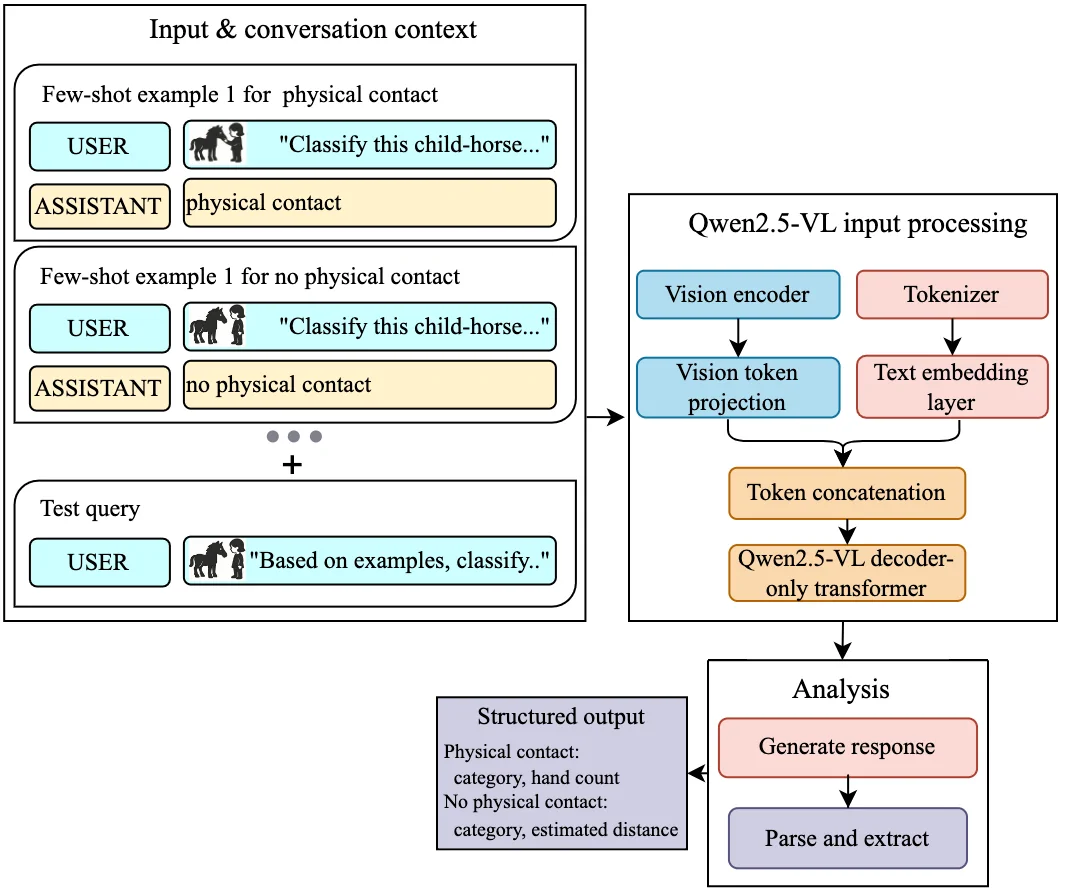
Pipeline of the proxemics analysis module for analyzing child-horse interaction images from the Yawardani Jan-ga equine-assisted learning program. A few-shot reference example and a query image are combined into a structured prompt, which is then processed by Qwen2.5-VL-7B. The output is parsed to extract the contact classification and relevant subattributes.

### Body Posture Analysis Module

We further adopt Qwen2.5-VL-7B-Instruct as the foundational VLM model for body posture analysis. In addition, we explore 2 complementary techniques inspired by MCoT reasoning [42] and self-consistency [43] to support more interpretable and stable analysis. MCoT uses a 2-stage reasoning process that encourages the model to first describe observable visual evidence before making predictions. The self-consistency approach improves robustness by identifying predictions that remain stable across multiple independent reasoning paths. Our method separates the analysis into a 2-stage inference process and repeats this process across multiple stochastic runs (K=5). For computational efficiency, each input image is encoded once, and the resulting visual embeddings are reused across all K runs, so that output variability is primarily driven by the stochastic text generation. In stage 1 (rationale generation), the model is prompted to describe observable posture elements, including head position, shoulder alignment, spine curvature, and stance, without assigning a label. A higher sampling temperature is used to encourage variation in the generated rationales across runs. In stage 2 (classification), the model is conditioned on both the image and the stage 1 rationale to classify the posture as “confident,” “not confident,” or “indifferent,” using a low temperature to promote stable classification. Figure 4 depicts the 2-stage prompts. By adopting this “describe-then-decide” workflow, practitioners can assess not only the final prediction but also the reasoning that supports it.

A majority vote across K runs is used to determine the final classification. To evaluate the model’s reliability, we also calculate a combined confidence score as the product of the majority-vote agreement rate (the proportion of K runs that agree with the majority-vote class) and the rationale coherence (measured using SentenceBERT all-mpnet-base-V2), to help practitioners identify low-confidence predictions that may require manual review. Figure 5 illustrates this parallel sampling and semantic verification process for body posture analysis.

**Figure 4:**
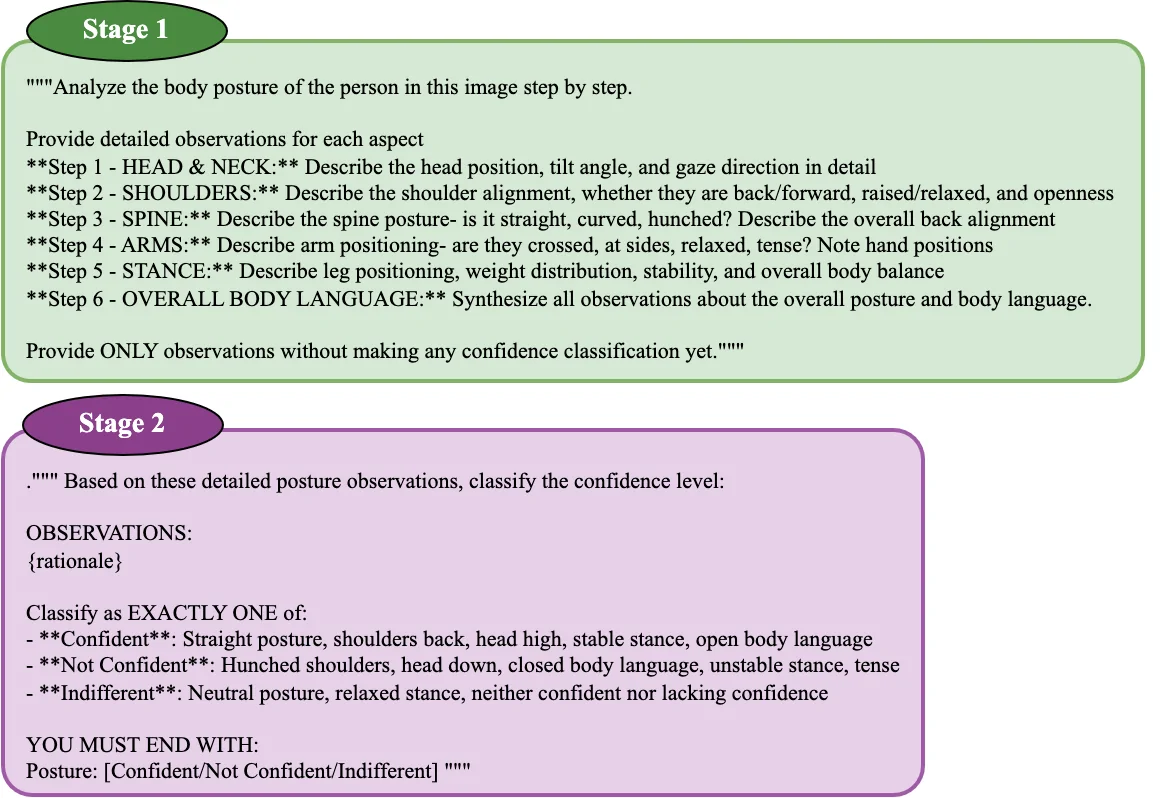
Two-stage multimodal chain-of-thought prompts used in the body posture analysis module for analyzing child-horse interaction images from the Yawardani Jan-ga equine-assisted learning program. Stage 1 (rationale generation) prompts Qwen2.5-VL-7B to describe observable posture elements, including head position, shoulder alignment, spine curvature, and stance, without assigning a classification label. Stage 2 (classification) conditions the model on both the image and the stage 1 rationale to classify body posture as “confident,” “not confident,” or “indifferent.”.

**Figure 5:**
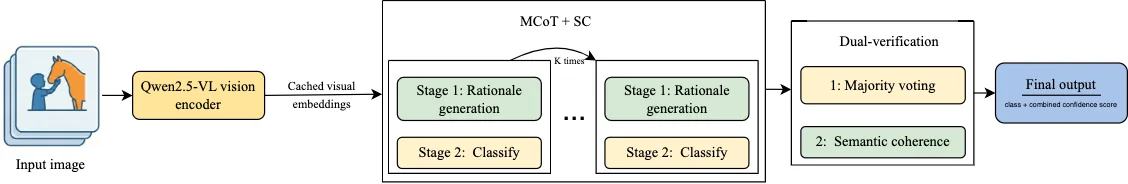
Pipeline of the body posture analysis module for analyzing child posture in child-horse interaction images from the Yawardani Jan-ga equine-assisted learning program. The diagram illustrates the parallel sampling and semantic verification process. For each input image, 2-stage multimodal chain-of-thought (MCoT) inference is repeated across K=5 independent runs with stochastic sampling. A majority vote determines the final posture classification, and rationale coherence is assessed using pairwise cosine similarity of SentenceBERT embeddings across runs to produce a combined confidence score. SC: self-consistency.

### Dataset and Preprocessing

#### Dataset

To develop and evaluate the proposed AI-assisted framework, we curated task-specific datasets derived from real images collected during Yawardani Jan-ga EAL sessions, in collaboration with certified EAL practitioners. The image datasets were drawn from sessions involving 10 participants (5 male and 5 female). Male participants ranged in age from 6 to 12 years, and female participants from 6 to 14 years. While the Yawardani Jan-ga program serves young people aged 6 to 25 years, the participants in this study fell within the younger age range due to the available and eligible data provided for analysis. The child participants included in the reference images and test images were mutually exclusive. Ground-truth labels were established through independent annotation by 2 raters from the expert panels. Both raters independently labeled all images in the activity classification and proxemics datasets. Interrater agreement was assessed using Cohen κ, yielding κ=0.938 for activity classification and κ=0.955 for proxemics classification. Disagreements were reviewed and resolved through discussion, with final consensus labels used as the ground-truth annotations. Across all modules, reference images were selected in collaboration with EAL practitioners to represent typical instances of each category. Selection was guided by whether each image reflected the intended category definition and could serve as a meaningful reference example for that category. The reference images were not selected through model-based optimization and were not chosen based on test-set performance.

The activity classification dataset was split into 3 subsets: training (36 reference images: 4 images × 9 categories) for few-shot learning and a separate pool of 464 images, which was split into a validation set (90 images, 20%) for hyperparameter optimization of the text-image fusion weight α, and a test set (374 images, 80%) for final evaluation. The test set comprises ball activities (n=52 images), grooming (n=50), herd observation (n=40), leading the horse (n=48), leading with obstacles (n=17), mounted sessions (n=35), painting (n=54), touching (n=47), and washing the horse (n=31). The reference, validation, and test sets contain images from 2, 3, and 5 child participants, respectively, and no child participant appears in more than one set. Some horses appear across sets due to the limited number of horses in the program.

The proxemics classification dataset was curated and annotated by the same expert panel, which categorized each image based on observable spatial relationships, including hand positioning, body contact points, and proximity indicators. The dataset comprises 268 images, divided into 2 groups: physical contact (n=108) and no physical contact (n=160). Two images from each category were reserved as reference examples for few-shot learning. For body posture analysis, we curated 193 images that provided sufficient visual clarity of posture-related features. Unlike the previous 2 datasets, this dataset does not contain categorical ground-truth labels. Instead, it was used to evaluate the reliability and consistency of the model’s analytical outputs under the proposed reasoning pipeline.

#### Data Preprocessing

The preprocessing steps are applied to images before feeding them into the AI models to ensure compatibility and optimal performance. All input images are standardized to RGB format. For activity classification using SigLIP2, images are resized to 384×384 pixels with default ImageNet normalization applied. For proxemics and body posture analysis using Qwen2.5-VL-7B, images are resized to 1024 pixels. For comparison models, CLIP uses 224×224 pixel resizing with ImageNet normalization.

#### Experimental Settings

For comparative models, SigLIP2 was evaluated against CLIP-ViT-L-14 under identical experimental conditions, with preprocessing adjusted to each model’s input resolution requirements. LLaVA-OneVision-7B was evaluated under identical prompt conditions, temperature, and token settings across all shot-learning paradigms, with images resized to 1024 pixels consistent with Qwen2.5-VL-7B preprocessing. For proxemics analysis, the temperature was set to τ=0.01 with a maximum output length of 512 tokens to promote deterministic outputs and to allow sufficient response length for structured reasoning. For body posture analysis, we used K=5 sampling runs per image with the temperature τ=0.7 (stage 1, maximum 512 tokens) to encourage diverse rationale generation, and τ=0.1 (stage 2, maximum 128 tokens) to promote stable classification. Semantic similarity between generated rationales was computed using SentenceBERT (all-mpnet-base-v2). To ensure evaluation consistency, the same prompt template was applied uniformly to all images, and no image-specific prompt adjustments were made during the analysis. All experiments were conducted on a desktop workstation equipped with an NVIDIA GeForce RTX 4090 GPU (24 GB VRAM), an Intel Core i9-14900F processor, and 32 GB RAM.

#### Evaluation Metrics

For activity classification and proxemics analysis, we used accuracy, precision, recall, and *F*_1_-score to evaluate performance against ground-truth labels. Precision, recall, and *F*_1_-score were macroaveraged, reducing the influence of class imbalance on the reported metrics. At the same time, accuracy was reported as the overall proportion of correctly classified images. For body posture analysis, we assessed the model reliability using internal consistency measures across multiple runs. First, we computed the agreement rate (*R*_agree_), a stability metric derived from the self-consistency framework [43]. It measures the proportion of images where the model yields identical predictions across N runs.



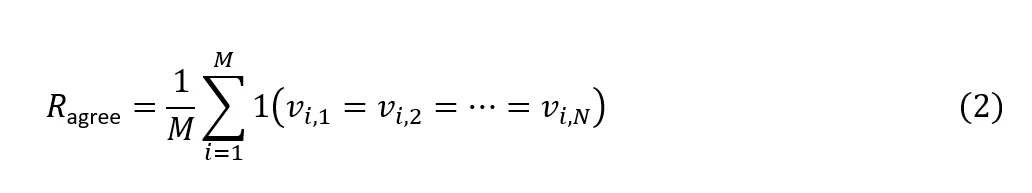



Where *M* is the total number of images in the dataset, *v_i,N_* is the prediction for image *i* at run N, and 1(.) serves as an indicator function that returns 1 when all predictions across all N runs are identical, and 0 otherwise.

Second, we calculated the average consistency (*C_avg_*) [43]:



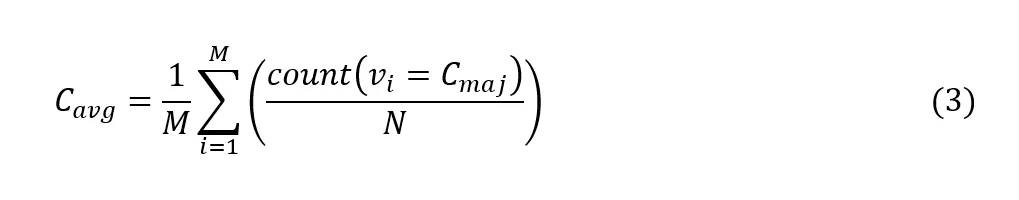



where *C_maj_* denotes the majority class determined for the image *i*, and the numerator counts how many runs predicted this majority label.

Third, we measured Rationale Coherence (*R_c_*). This is the average pairwise cosine similarity between SentenceBERT embeddings of the generated rationales [49,50].



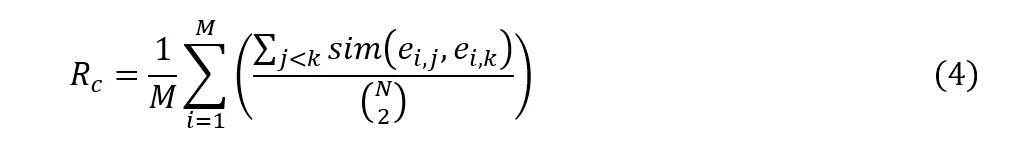



Where *e_i,j_* represents the embedding of the rationale for the image *i* during run *j*, and *sim*(.) denotes the cosine similarity function.

Finally, we calculated vote entropy (*H*) [51] to quantify predictive uncertainty:



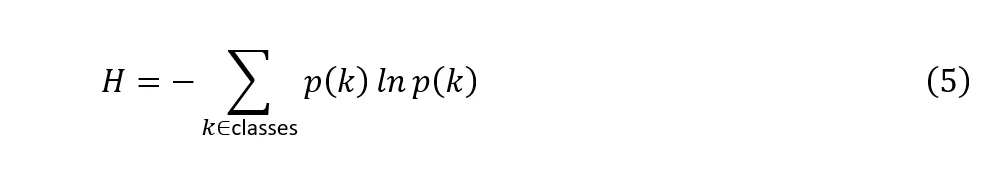



where *p*(*k*) is the empirical probability of class *k* across runs. A value of *H*=0 implies perfect agreement, while higher values reflect greater prediction divergence.

### Ethical Considerations

Ethics approval for this study was obtained from the Western Australian Aboriginal Health Ethics Committee (WAAHEC; HREC926). This study used deidentified data collected through the original Yawardani Jan-ga program and evaluation. Participants, or their parents/carers, where required, provided informed consent for the original data collection and for their deidentified data to be used for approved research purposes, including secondary analysis. Young people were also asked for assent or consent in an age-appropriate way. No additional consent was required for this secondary analysis. Participant privacy and confidentiality were protected throughout the study. The dataset did not contain directly identifiable personal information, and all analyses were conducted using deidentified records. No images or supplementary materials included in the manuscript allow identification of individual participants. No additional participant compensation was provided for this secondary analysis.

## Results

### Activity Classification Performance

We evaluated the activity classification module on the curated dataset and compared its performance with the CLIP model as a baseline. To assess the effect of contextual evidence, we examined 0-shot through 4-shot configurations. The results are summarized in Table 1.

**Table 1:** Activity classification performance of Sigmoid Loss for Language-Image Pre-training 2 (SigLIP2) and CLIP-ViT-L-14 for classifying 9 therapeutic activities in child-horse interaction images from the labeled activity classification dataset curated by the Yawardani Jan-ga equine-assisted learning program. Results are reported across 0-shot to 4-shot learning configurations; all performance metrics are reported as percentages and macroaveraged where applicable.

Learning setting and model		Accuracy (%)	Macro precision (%)	Macro recall (%)	Macro *F*_1_-score (%)
**0-shot**					
	SigLIP2	89.3	90.4	89.7	89.6
	CLIP^a^	57.5	64.9	57.6	52.5
**1-shot**					
	SigLIP2	90.4	90.8	91.3	90.7
	CLIP	61.8	64.2	61.5	60.6
**2-shot**					
	**SigLIP2**	90.6	92.0	91.1	91.1
	CLIP	62.0	66.2	63.2	61.6
**3-shot**					
	SigLIP2	89.8	90.2	90.8	90.0
	CLIP	62.3	68.5	62.7	61.3
**4-shot**					
	SigLIP2	90.6	91.8	91.3	91.2
	CLIP	61.5	63.4	62.4	60.9

^a^CLIP: Contrastive Language-Image Pre-training.

Across all shot settings, SigLIP2 outperformed CLIP across all evaluation metrics, with macro *F*_1_-score performance gaps ranging from 28.7% to 37.1% (Table 1). SigLIP2 macro *F*_1_-score ranged from 89.6% in the 0-shot configuration to 91.2% in the 4-shot configuration. CLIP performance remained substantially lower across all settings, ranging from 52.5% to 61.6% macro *F*_1_-score. Although SigLIP2 4-shot achieved a slightly higher macro *F*_1_-score, 2-shot was selected because it achieved the same accuracy with fewer reference images. Because SigLIP2 and CLIP-ViT-L-14 were evaluated on the same test images, a McNemar test with continuity correction was used to assess whether their paired classification errors differed significantly across all learning configurations. As shown in Table 2, SigLIP2 significantly outperformed CLIP-ViT-L-14 across all settings (*P*<.001).

**Table 2:** McNemar test (with continuity correction) comparing paired activity classification outcomes of Sigmoid Loss for Language-Image Pre-training 2 (SigLIP2) and CLIP-ViT-L-14 across all learning configurations on the same test images.

Learning setting	n_01_^a^	n_10_^b^	Chi-square (*df*)	*P* value
0-shot	134	15	93.45 (1)	<.001
1-shot	122	15	82.01 (1)	<.001
2-shot	124	17	79.69 (1)	<.001
3-shot	118	15	78.23 (1)	<.001
4-shot	126	17	81.57 (1)	<.001

^a^n_01_=number of images correctly classified by SigLIP2 but misclassified by Contrastive Language-Image Pre-training (CLIP).

^b^n_10_=number of images misclassified by SigLIP2 but correctly classified by CLIP.

The category-level confusion matrix performance is detailed in Figures 6 and 7. SigLIP2 diagonal values ranged from 0.74 to 1.00 across categories, with perfect classification for “ball” and “washing.” CLIP diagonal values ranged from 0.00 to 0.94, with particularly low performance for “leading the horse” at 0.00, “leading with obstacles” at 0.59, and “painting” at 0.44.

On the validation set under the 2-shot configuration, performance for both models increased as the text weight α increased. SigLIP2 achieved peak performance at α=.9, while CLIP peaked at α=.8 (Figure 8). Consequently, these optimal weights were applied during the final test set evaluation.

**Figure 6:**
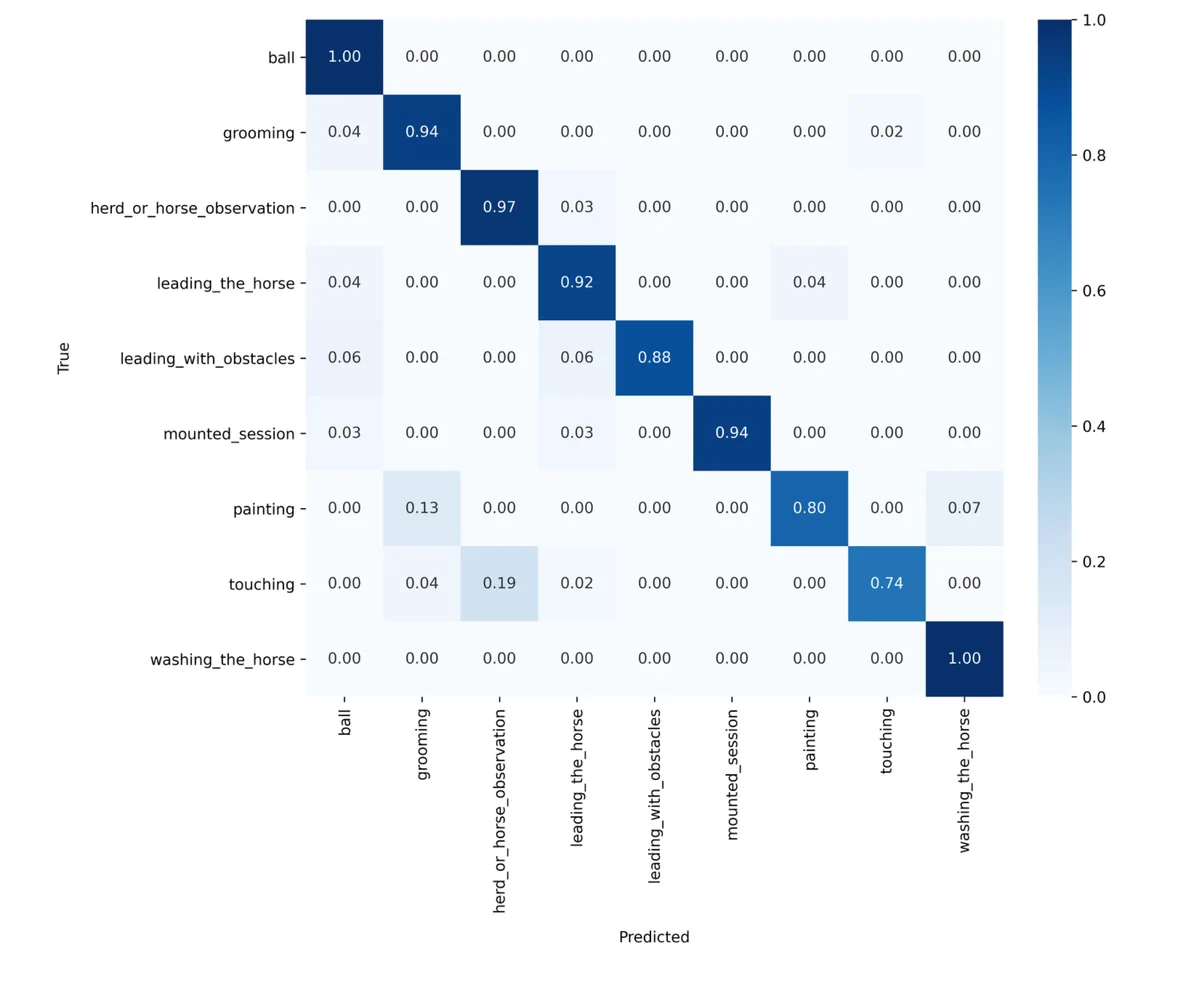
Confusion matrix for Sigmoid Loss for Language-Image Pre-training 2 under the 2-shot learning configuration for classifying 9 therapeutic activities in child-horse interaction images. Rows indicate true activity labels and columns indicate predicted labels.

**Figure 7:**
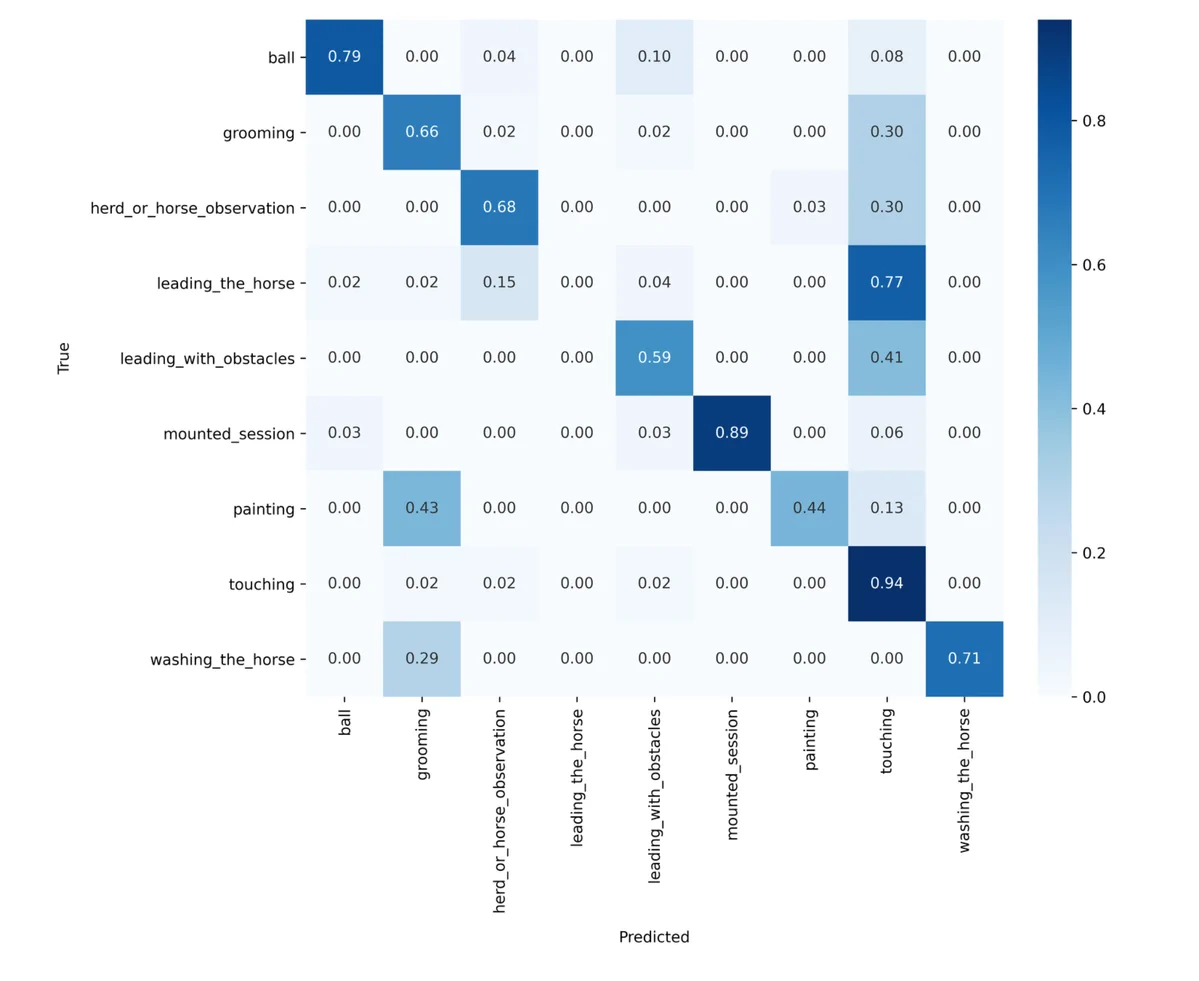
Confusion matrix for CLIP-ViT-L-14 under the 2-shot learning configuration for classifying 9 therapeutic activities in child-horse interaction images. Rows indicate true activity labels and columns indicate predicted labels.

**Figure 8:**
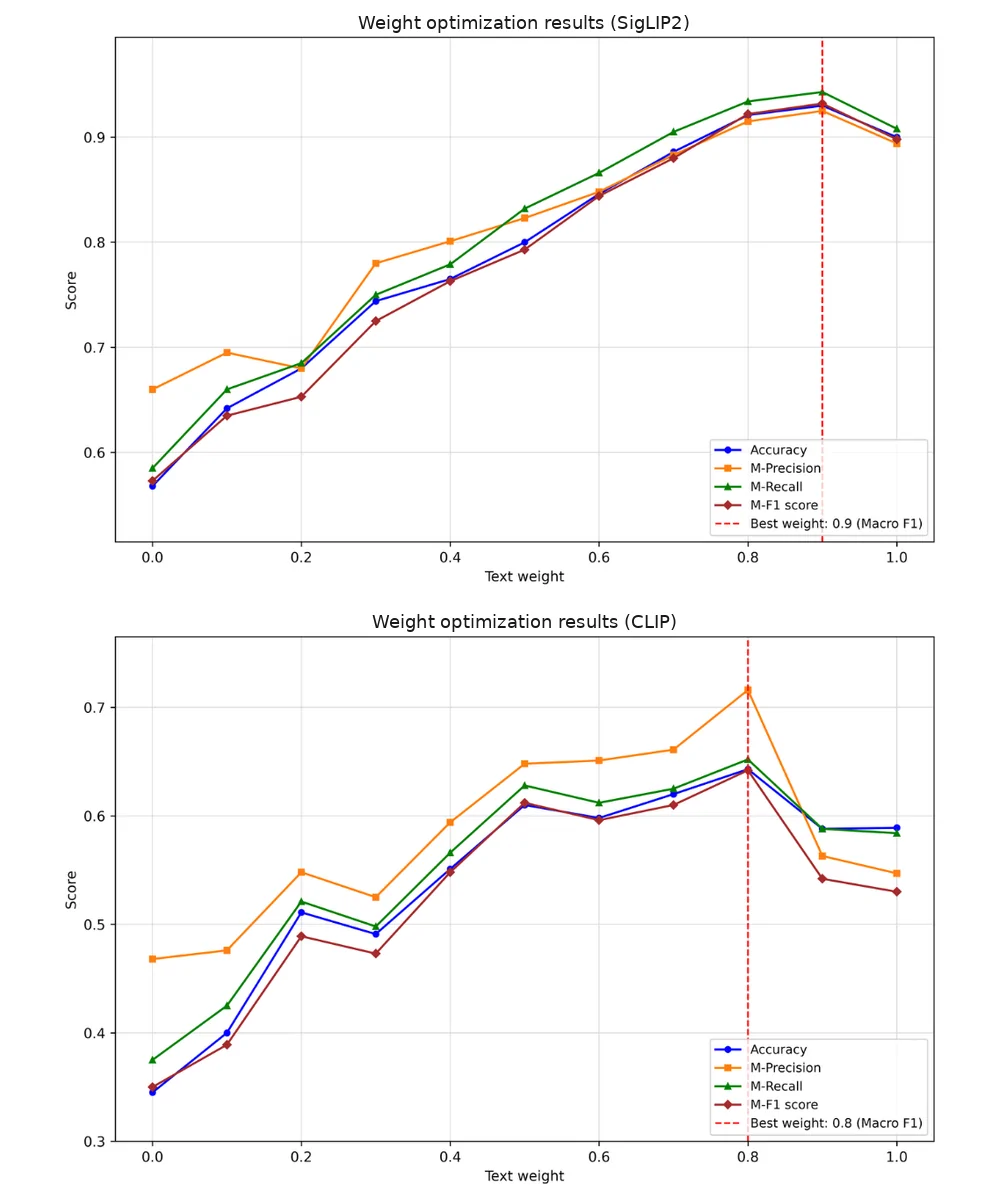
Effect of varying the text-image fusion weight (α) on activity classification performance for Sigmoid Loss for Language-Image Pre-training 2 (SigLIP2) and CLIP-ViT-L-14 on the same validation set. Macro F1-score is reported for each α value. The weight α controls the relative contribution of textual similarity (α) and visual prototype similarity (1–α), with values varied from 0.0 to 1.0.

### Proximity Classification Evaluation

We benchmarked our approach against the LLaVA-OneVision-7B model on the test dataset across all shot learning configurations, as detailed in Table 3. Qwen2.5-VL-7B outperformed LLaVA-OneVision across all learning configurations. Qwen2.5-VL-7B achieved its best macro *F*_1_-score of 88.5% in the 1-shot configuration, with an accuracy of 88.6%, macro precision of 89.0%, and macro recall of 88.0%. In the 2-shot configuration, accuracy was 87.9%, macro precision was 87.0%, macro recall was 87.5%, and macro *F*_1_-score was 87.3%. LLaVA-OneVision’s accuracy was 80.3% at 0-shot and 65.5% at 2-shot, with macro *F*_1_-scores of 78.3% and 53.9%, respectively. McNemar test with continuity correction was applied to assess whether the paired classification errors between Qwen2.5-VL-7B and LLaVA-OneVision differed significantly across all learning configurations. As shown in Table 4, Qwen2.5-VL-7B significantly outperformed LLaVA-OneVision across all settings (*P*=.01 for 0-shot and *P*<.001 for few-shot configurations).

To further examine how each model behaves at the individual-class level, Table 5 presents per-category performance for both “physical contact” and “no physical contact” across all learning methods. For Qwen2.5-VL-7B, 0-shot learning yielded a physical contact recall of 72.0% and an *F*_1_-score of 82.3%, which shifted to 85.0% recall and 86.0% *F*_1_-score under 1-shot learning. The “no physical contact” class achieved 91.0% recall and 91.0% *F*_1_-score in the 1-shot setting. Under 2-shot learning, physical contact metrics were 84.0% precision, 86.0% recall, and 85.0% *F*_1_-score, while no physical contact metrics were 90.0% precision, 89.0% recall, and 89.5% *F*_1_-score.

**Table 3:** Overall proxemics classification performance of Qwen2.5-VL-7B and LLaVA-OneVision-qwen2-7b for classifying physical contact in child-horse interaction images from the Yawardani Jan-ga equine-assisted learning program. Results are reported across 0-shot to 2-shot learning configurations on the same 264 test images; all performance metrics are reported as percentages and macroaveraged where applicable.

Learning setting and model		Accuracy (%)	Macro precision (%)	Macro recall (%)	Macro *F*_1_-score (%)
**0-shot**					
	Qwen2.5-VL-7B	87.5	90.0	85.0	86.4
	LLaVA-OneVision	80.3	83.2	76.9	78.3
**1-shot**					
	Qwen2.5-VL-7B	88.6	89.0	88.0	88.5
	LLaVA-OneVision	68.2	74.0	61.2	59.1
**2-shot**					
	Qwen2.5-VL-7B	87.9	87.0	87.5	87.3
	LLaVA-OneVision	65.5	72.0	58.0	53.9

**Table 4:** McNemar test (with continuity correction) comparing paired proxemics classification outcomes of Qwen2.5-VL-7B and LLaVA-OneVision across all learning configurations on the same test images. n01 = number of images correctly classified by Qwen2.5-VL-7B but misclassified by LLaVA-OneVision; n10 = number of images misclassified by Qwen2.5-VL-7B but correctly classified by LLaVA-OneVision.

Learning setting	n_01_	n_10_	Chi-square (*df*)	*P* value
0-shot	36	17	6.11 (1)	.01
1-shot	67	13	35.11 (1)	<.001
2-shot	73	14	38.67 (1)	<.001

We also note that, unlike Qwen2.5-VL-7B, LLaVA-OneVision showed reduced stability when additional reference examples were provided. For LLaVA-OneVision, the “physical contact” *F*_1_-score dropped from 70.9% in the 0-shot setting to 39.9% and 30.7% in the 1-shot and 2-shot settings, respectively, with recall falling from 59.4% to 19.0%. In contrast, “no physical contact” recall remained above 94% across all settings. The *F*_1_-score gap between the 2 classes widened from 14.7% in the 0-shot setting to 46.4% in the 2-shot setting, suggesting that predictions became increasingly biased toward the majority class as more reference examples were added. This pattern may reflect difficulty in consistently using additional visual reference examples for fine-grained proximity classification. Similar 0-shot to few-shot performance declines have been reported for LLaVA-family models [52], while stronger few-shot stability has been observed in larger multimodal models such as Qwen2.5-VL-32B [53]. Our findings are consistent with these reports and further indicate that, at the 7B scale, Qwen2.5-VL-7B remained more stable than LLaVA-OneVision-7B on this task.

**Table 5:** Per-category proxemics classification performance for Qwen2.5-VL-7B and LLaVA-OneVision across 0-shot to 2-shot learning configurations.

Learning setting, model, and category			Precision (%)	Recall (%)	*F*_1_-score (%)
**0-shot**					
	**Qwen2.5-VL-7B**				
		Physical contact	96.0	72.0	82.3
		No physical contact	84.0	98.0	90.5
	**LLaVA-OneVision**				
		Physical contact	88.0	59.4	70.9
		No physical contact	78.4	94.3	85.6
**1-shot**					
	**Qwen2.5-VL-7B**				
		Physical contact	87.0	85.0	86.0
		No physical contact	91.0	91.0	91.0
	**LLaVA-OneVision**				
		Physical contact	82.0	26.4	39.9
		No physical contact	66.0	96.0	78.2
**2-shot**					
	**Qwen2.5-VL-7B**				
		Physical contact	84.0	86.0	85.0
		No physical contact	90.0	89.0	89.5
	**LLaVA-OneVision**				
		Physical contact	80.0	19.0	30.7
		No physical contact	64.0	97.0	77.1

### Body Posture Analysis Evaluation

To evaluate model reliability, we compared our full model, Qwen2.5-VL-7B + MCoT + SC, with 2 baseline inference strategies using 4 internal consistency metrics. Three strategies were evaluated: (1) single-stage CoT (simple CoT), which generates the reasoning and classification in a single pass; (2) multimodal 2-stage CoT (MCoT), which first produces a descriptive visual rationale and then conditions the final classification on both the image and the generated rationale; and (3) MCoT with self-consistency (MCoT+SC), which further aggregates multiple MCoT inference paths using majority voting to obtain a consensus prediction. Each method was evaluated across 5 independent experimental repeats. Within each repeat, the MCoT+SC strategy performed K=5 stochastic inference paths for each image. Internal consistency metrics were computed for each image and then summarized across the test set. The reported means and SDs were calculated across the 5 independent experimental repeats.

As shown in Table 6, simple CoT achieved a rationale coherence score of 0.913, an agreement rate of 0.104, and a vote entropy of 0.653 across 5 independent runs. The MCoT approach improved output consistency, with an agreement rate of 0.528, vote entropy of 0.300, and a rationale coherence score of 0.931. The MCoT+SC approach achieved the highest agreement rate of 0.705 and the lowest vote entropy of 0.174, indicating more stable classification outputs across runs, although its rationale coherence score was lower (0.857) than MCoT (0.931).

**Table 6:** Evaluation of reasoning stability and consistency for the body posture analysis module across 3 inference strategies: simple chain-of-thought (CoT), multimodal chain-of-thought (MCoT), and MCoT with self-consistency (SC; MCoT+SC), each assessed over 5 independent experimental repeats on 193 images.

Metrics	Qwen2.5-VL-7B + simple CoT, mean (SD)	Qwen2.5-VL-7B + MCoT, mean (SD)	Qwen2.5-VL-7B + MCoT+SC (our model), mean (SD)
Average consistency	0.675 (0.006)	0.853 (0.004)	0.916 (0.010)
Agreement rate (*R*_agree_)	0.104 (0.004)	0.528 (0.019)	0.705 (0.024)
Vote entropy (*H*)	0.653 (0.009)	0.300 (0.010)	0.174 (0.027)
Rationale coherence (*R*_*c*_)	0.913 (0.001)	0.931 (0.001)	0.857 (0.002)

It should be noted that the consistency metrics reported here assess the stability of the model’s outputs, not the factual correctness of the posture classifications. Without ground-truth posture annotations, these metrics should be understood as indicators of reliability rather than accuracy. Validation against expert-annotated labels remains essential before this module can be used in clinical applications.

## Discussion

### Principal Findings

The evaluation of the AI-assisted VLM framework suggests that few-shot learning may support the classification of observable activities and spatial interactions in EAL settings using limited labeled data. For activity classification, SigLIP2 achieved stronger overall performance than CLIP-ViT-L-14 across the evaluated settings, suggesting that the choice of vision-language backbone is important for recognizing EAL activity categories in specific settings. The validation-based optimization of the text-image fusion weight (α) suggested that textual activity descriptions contributed useful discriminative information alongside visual prototypes within the proposed fusion strategy. The peak α = 0.9 for SigLIP2 indicates that the model relies predominantly on well-defined textual descriptions, where carefully specified activity definitions provide a strong discriminative signal for classification. The visual prototypes derived from reference images play a complementary role and may offer additional contextual grounding, altogether improving performance over the 0-shot setting. In the proxemics classification task, Qwen2.5-VL-7B showed relatively stable performance across learning configurations, whereas LLaVA-OneVision performed less consistently when additional examples were provided. For body posture analysis, the MCoT+SC approach improved average consistency, agreement rate, and vote entropy, indicating more stable classification outputs across repeated runs. However, its rationale coherence score was lower. This may be partly due to the higher sampling temperature used during rationale generation, which was intended to encourage diverse reasoning paths across runs but may also reduce semantic similarity among the generated rationales.

The framework may provide complementary support for documenting observable EAL interactions. The activity classification module identifies the activity type and provides a confidence score, which may help practitioners organize session records according to activity categories. The proxemics module classifies whether physical contact is present, providing one observable indicator of child-horse spatial interaction patterns. The body posture module classifies observable postural states and provides a natural-language rationale describing the posture elements that informed the classification, which may improve transparency in how the model reached its output. Together, these outputs may complement practitioner-led observational notes and support a more structured review of interaction patterns over time. These findings should not be interpreted as evidence that the framework can assess therapeutic progress or clinical outcomes; such applications would require further validation against practitioner ratings, participant goals, and standardized well-being or therapeutic outcome measures.

### Limitations

Several limitations of this study warrant discussion. First, the availability of large, diverse datasets in this specialized EAL context remains limited, and this study relied on relatively small real-world datasets. Expanding dataset size and diversity may improve generalizability. Second, the McNemar test assumes independent paired observations, but the dataset includes multiple images from the same participants, which may partially violate this assumption, potentially affecting the reported significance. Third, image quality, occlusion, and visually complex outdoor environments, which are possible in real-world EAL sessions, may affect performance across all 3 modules. Fourth, the body posture module lacks ground-truth labels, and model reliability was assessed through internal consistency measures rather than supervised evaluation. These metrics capture output stability but should not be interpreted as evidence of classification accuracy. Fifth, the framework operates on static images rather than continuous video and therefore does not capture dynamic interaction patterns such as posture transitions or evolving engagement. Sixth, the framework primarily analyses the child’s observable behaviors and captures only limited aspects of the horse’s responses. Although the proxemics module describes child-horse spatial interaction by estimating distance categories during noncontact interactions, deriving accurate spatial measurements from a single uncalibrated image remains technically challenging. Seventh, the body posture module is the most computationally demanding component, as it has 5 inference runs per image to support self-consistency, which may increase processing latency and limit practical deployment in remote service areas with constrained infrastructure. Eighth, the pretrained VLMs used in this study were trained on generalized, large-scale datasets and may carry culturally specific assumptions about body language and nonverbal behavior. This is particularly important for the body posture module, where classifications such as “confident” or “not confident” may depend on culturally specific visual cues that may not have the same meaning across communities. Therefore, body posture outputs should be interpreted as exploratory analytical outputs rather than culturally validated assessments, and reviewed cautiously alongside practitioner judgment, cultural knowledge, and the broader therapeutic context. Ninth, the framework relies on precise textual descriptions that draw sharp boundaries between activities, such as defining grooming as involving tools and touching as involving bare hands. While this design supports consistent annotation and evaluation, interactions in practice are fluid and may involve overlapping or transitional behaviors. Since the framework assigns a single label per image, it does not capture cases where 2 activities co-occur or when a child transitions between them. Finally, although the same prompt template was consistently applied to all images in this study, systematic testing of multiple prompt formulations was beyond our scope.

### Future Directions

Several directions for future work follow from these limitations. First, future work should prioritize developing expert-annotated posture datasets to enable supervised evaluation of prediction performance. Larger and more participant-diverse datasets would support more robust performance evaluation and statistical analysis. Depth-aware or multiview approaches could also be explored to provide more reliable estimates of child-horse distance and spatial positioning. More efficient deployment strategies should be investigated, such as quantization, model distillation, reduced self-consistency sampling, and smaller, locally deployable models. Culturally informed adaptation and validation should be prioritized in collaboration with Aboriginal communities and EAL practitioners, including co-designing posture categories and interpretive criteria before their therapeutic application. Performance on more ambiguous interactions may differ from the results reported here, and future work could investigate multilabel classification, uncertainty-aware outputs, and temporal analysis across image sequences to better capture the dynamic nature of EAL interactions. Prompt robustness and the degree to which variations in wording may influence classification outputs should also be examined. Finally, future work should expand expert-annotated datasets, develop video-based analysis of EAL interactions, incorporate horse behavioral responses, and conduct clinical validation to examine whether framework outputs align with practitioner ratings and standardized therapeutic or well-being outcome measures.

### Conclusions

This study proposed a VLM-based AI framework for EAL settings, comprising 3 modules: activity classification, proxemics analysis, and body posture analysis. The findings suggest that AI- and VLM-based approaches have the potential to support the analysis of images captured during EAL sessions. This study represents an initial step toward more structured documentation and analysis of observable child-horse therapeutic interactions in EAL settings. Such digital health tools may reduce practitioner burden, complement practitioner expertise and clinical judgment, and facilitate data-driven research into equine-assisted interventions.

## Abbreviations

CLIP: Contrastive Language-Image Pre-training
CoT: chain of thought
EAL: equine-assisted learning
LLaVA: Large Language-and-Vision Assistant
MCoT: multimodal chain of thought
SC: self-consistency
SigLIP: Sigmoid Loss for Language-Image Pre-training
VLM: vision-language model

## Data Availability

The datasets analyzed during this study are not publicly available due to data governance considerations. Reasonable requests for access may be directed to the corresponding authors, subject to relevant ethics approvals, Aboriginal-led governance processes and appropriate data access agreements.

The inference scripts, prompt templates, and data processing pipelines used in this study are available from the corresponding authors upon reasonable request.

## References

[1] Kendall E, Maujean A, Pepping CA, Downes M, Lakhani A, Byrne J, Macfarlane K (2015). A systematic review of the efficacy of equine-assisted interventions on psychological outcomes. Eur J Psychother Couns.

[2] Carlsson I, Bräutigam Ewe M, Nymberg P, Jormfeldt H (2024). Building up bit by bit, parent's experiences of equine-assisted intervention among children and adolescents with mental illness: a grounded theory study. Int J Qual Stud Health Well-being.

[3] Punzo K, Skoglund M, Carlsson I, Jormfeldt H (2022). Experiences of an equine-assisted therapy intervention among children and adolescents with mental illness in Sweden - a nursing perspective. Issues Ment Health Nurs.

[4] Ayala MD, Carrillo A, Iniesta P, Ferrer P (2021). Pilot study of the influence of equine assisted therapy on physiological and behavioral parameters related to welfare of horses and patients. Animals (Basel).

[5] Cagle-Holtcamp K, Nicodemus MC, Parker J, Dunlap MH (2019). Does equine assisted learning create emotionally safe learning environments for at-risk youth?. Journal of Youth Development.

[6] Frederick KE, Ivey Hatz J, Lanning B (2015). Not just horsing around: the impact of equine-assisted learning on levels of hope and depression in at-risk adolescents. Community Ment Health J.

[7] Scopa C, Contalbrigo L, Greco A, Lanatà A, Scilingo EP, Baragli P (2019). Emotional transfer in human-horse interaction: new perspectives on equine assisted interventions. Animals (Basel).

[8] (2023). Yawardani Janga. Yawardani Jan-ga.

[9] Coffin J (2019). The nguudu barndimanmanha project-improving social and emotional wellbeing in Aboriginal youth through equine assisted learning. Front Public Health.

[10] Coffin J, Vaz S, Kickett-Tucker C, Milroy H, Olsson C, Kirby M, Nelson L, McPhee R, Cross D (2024). Protocol to implement and evaluate a culturally secure, strength-based, equine-assisted learning program, "Yawardani Jan-ga" (horses helping), to support the social and emotional wellbeing of Australian Aboriginal children and young people. PLoS One.

[11] Hemingway A, Carter S, Callaway A, Kavanagh E, Ellis S (2019). An exploration of the mechanism of action of an equine-assisted intervention. Animals (Basel).

[12] Callara AL, Scopa C, Contalbrigo L, Lanatà A, Scilingo EP, Baragli P, Greco A (2024). Unveiling directional physiological coupling in human-horse interactions. iScience.

[13] Yorke J, Nugent W, Strand E, Bolen R, New J, Davis C (2012). Equine-assisted therapy and its impact on cortisol levels of children and horses: a pilot study and meta-analysis. Early Child Dev Care.

[14] Stolz I, Tillmann V, Anneken V, Froboese I (2022). Development of an ICF-based assessment tool for equine-assisted therapy: model structure and reliability. Eur J Phys Rehabil Med.

[15] Serpell J, McCune S, Gee N, Griffin JA (2017). Current challenges to research on animal-assisted interventions. Appl Dev Sci.

[16] Jibilian CA (2022). Impacts of Equine-Assisted Therapy Depicted Through Photography.

[17] Zeng D, Liao M, Tavakolian M, Guo Y, Zhou B, Hu D Deep learning for scene classification: a survey. arXiv.

[18] Zou Z, Chen K, Shi Z, Guo Y, Ye J (2023). Object detection in 20 years: a survey. Proc IEEE.

[19] Kwok WH, Zhang Y, Wang G (2024). Artificial intelligence in perinatal mental health research: a scoping review. Comput Biol Med.

[20] Zhang J, Huang J, Jin S, Lu S (2024). Vision-language models for vision tasks: a survey. IEEE Trans Pattern Anal Mach Intell.

[21] Hartsock I, Rasool G (2024). Vision-language models for medical report generation and visual question answering: a review. Front Artif Intell.

[22] Li C, Wong C, Zhang S, Usuyama N, Liu H, Yang J (2023). LLaVA-med: training a large language-and-vision assistant for biomedicine in one day. https://proceedings.neurips.cc/paper_files/paper/2023/file/5abcdf8ecdcacba028c6662789194572-Paper-Datasets_and_Benchmarks.pdf.

[23] Alarcon N (2018). Using AI to monitor horse safety. NVIDIA Technical Blog.

[24] Radford A, Kim JW, Hallacy C, Ramesh A, Goh G, Agarwal S (2021). Learning transferable visual models from natural language supervision. https://proceedings.mlr.press/v139/radford21a.html.

[25] Jia C, Yang Y, Xia Y, Chen YT, Parekh Z, Pham H (2021). Scaling up visual and vision-language representation learning with noisy text supervision. https://proceedings.mlr.press/v139/jia21b.html.

[26] Tschannen M, Gritsenko A, Wang X, Naeem M, Alabdulmohsin I, Parthasarathy N SigLIP 2: multilingual vision-language encoders with improved semantic understanding, localization, and dense features. arXiv.

[27] Zhai X, Mustafa B, Kolesnikov A, Beyer L (2023). Sigmoid loss for language image pre-training.

[28] Liu H, Li C, Wu Q, Lee Y (2023). Visual instruction tuning. https://proceedings.neurips.cc/paper_files/paper/2023/file/6dcf277ea32ce3288914faf369fe6de0-Paper-Conference.pdf.

[29] Bai S, Chen K, Wang J, Ge W, Song S Qwen2.5-VL technical report. arXiv.

[30] Bai J, Bai S, Yang S, Wang S, Tan S, Wang P A versatile vision-language model for understanding, localization, text reading, and beyond. arXiv.

[31] An X, Xie Y, Yang K, Zhang W, Zhao X, Cheng Z LLaVA-oneVision-1.5: fully open framework for democratized multimodal training. arXiv.

[32] Li B, Zhang Y, Guo D, Zhang R, Li F, Zhang H (2025). LLaVA-OneVision: Easy Visual Task Transfer. OpenReview.

[33] Giannone C, Dalla CE, Maccario C, Atallah E, Bovo M (2025). Recognition of horse behaviours using deep learning techniques. https://hdl.handle.net/11585/1015991.

[34] Galimzianov D, Vyshegorodtsev V, Nezhivykh I (2026). Monitoring horses in stalls: from object to event detection. Doklady Mathematics.

[35] Dalla Costa E, Bovo M (2025). Editorial: advances in the application of technology for monitoring horse welfare and health. Front Vet Sci.

[36] Schick T, Schütze H (2021). Exploiting cloze-questions for few-shot text classification and natural language inference.

[37] Brown T, Mann B, Ryder N, Subbiah M, Kaplan J, Dhariwal P (2020). Language models are few-shot learners. https://proceedings.neurips.cc/paper_files/paper/2020/file/1457c0d6bfcb4967418bfb8ac142f64a-Paper.pdf.

[38] Pachetti E, Colantonio S (2024). A systematic review of few-shot learning in medical imaging. Artif Intell Med.

[39] Ferber D, Wölflein G, Wiest IC, Ligero M, Sainath S, Ghaffari Laleh N, El Nahhas OSM, Müller-Franzes G, Jäger D, Truhn D, Kather JN (2024). In-context learning enables multimodal large language models to classify cancer pathology images. Nat Commun.

[40] Agbareia R, Omar M, Zloto O, Glicksberg BS, Nadkarni GN, Klang E (2025). Multimodal LLMs for retinal disease diagnosis via OCT: few-shot versus single-shot learning. Ther Adv Ophthalmol.

[41] Wei J, Wang X, Schuurmans D, Bosma M, Chi E, Le Q (2022). Chain of thought prompting elicits reasoning in large language models. https://proceedings.neurips.cc/paper_files/paper/2022/file/9d5609613524ecf4f15af0f7b31abca4-Paper-Conference.pdf.

[42] Zhang Z, Zhang A, Li M, Zhao H, Karypis G, Smola A Multimodal chain-of-thought reasoning in language models. OpenReview.

[43] Wang X, Wei J, Schuurmans D, Le Q, Chi E, Zhou D (2023). Self-consistency improves chain of thought reasoning in language models. https://openreview.net/forum?id=1PL1NIMMrw.

[44] Faria FTJ, Baniata LH, Choi A, Kang S (2025). Analyzing diagnostic reasoning of vision–language models via zero-shot chain-of-thought prompting in medical visual question answering. Mathematics.

[45] Wang Y, Liu J, Gao S, Feng B, Tang Z, Gai X (2025). V2t-cot: from vision to text chain-of-thought for medical reasoning and diagnosis.

[46] Le-Duc K, Nguyen D, Trinh P, Nguyen T, Diep N, Ngo A S-chain: structured visual chain-of-thought for medicine. arXiv.

[47] Anis AM, Ali H, Sarfraz S On the limitations of vision-language models in understanding image transforms. arXiv.

[48] Wang F, Zhang Z, Zhang X, Wu Z, Mo T, Lu Q, Wang W, Li R, Xu J, Tang X, He Q, Ma Y, Huang M, Wang S (2025). A comprehensive survey of small language models in the era of large language models: techniques, enhancements, applications, collaboration with LLMs, and trustworthiness. ACM Trans Intell Syst Technol.

[49] Salton G, Wong A, Yang CS (1975). A vector space model for automatic indexing. Commun ACM.

[50] Manakul P, Liusie A, Gales M (2023). Selfcheckgpt: zero-resource black-box hallucination detection for generative large language models.

[51] Engelson S, Dagan I (1996). Minimizing manual annotation cost in supervised training from corpora.

[52] Xia P, Zhu K, Li H, Wang T, Shi W, Wang S (2025). MMed-RAG: versatile multimodal RAG system for medical vision language models. https://proceedings.iclr.cc/paper_files/paper/2025/hash/a559a5a8aa5ae6682ced009ad97cdb16-Abstract-Conference.html.

[53] Komanduri A, Bhaila K, Wu X (2025). CausalVLBench: benchmarking visual causal reasoning in large vision-language models.

